# Global Research Landscapes and Emerging Trends in Music Therapy for Older Adults: A Comprehensive Bibliometric Analysis

**DOI:** 10.12688/f1000research.187349.1

**Published:** 2026-08-24

**Authors:** Kanjanee Phanphairoj, Sutthisan Chumwichan, Ratsiri Thato, Dneya Udtaisuk, Debby Syahru Romadlon, Patcharin Krongtham, Faizul Hasan

**Affiliations:** 1 Faculty of Nursing, Chulalongkorn University, Bangkok, Bangkok, Thailand; 2 Multidisciplinary approaches to improve holistic well-being for all ages: Harmonizing nursing science and music Research Group, Chulalongkorn University, Bangkok, Bangkok, Thailand; 3 Invited Researcher, Faculty of Education, Chulalongkorn University, Bangkok, Bangkok, Thailand; 4 Faculty of Education, Chulalongkorn University, Bangkok, Bangkok, Thailand

**Keywords:** Music Therapy; Geriatric Care; Dementia; Bibliometric Analysis; Nonpharmacological Intervention.

## Abstract

**Background:**

Music therapy is a crucial nonpharmacological intervention in geriatric care that can improve cognitive function, emotional well-being, and adjustment during transitions to long-term care. However, several research gaps, including variability in study designs, limited evidence regarding long-term efficacy, and unclear mechanisms of action, limit such therapy’s systematic implementation. We performed bibliometric analysis to examine global research on music therapy involving older adults and identify major contributors, collaboration networks, and key themes in this area of research.

**Methods and findings:**

A comprehensive search of the Scopus database was conducted to identify relevant articles published from inception through February 2026. We used bibliometric methods to analyze annual publication trends, geographic and institutional productivity, international collaboration patterns, author influence, and keyword co-occurrence. Data visualization and network mapping were performed using VOSviewer and the Bibliometrix package in R. The analysis included 5253 publications. China had the highest publication output (705), followed by the United States (675) and the United Kingdom (309). Italy had the highest level of international collaboration (multiple country publication ratio: 31.5). The University of Toronto was the most productive institution (92 publications). Coauthorship analysis identified six international collaboration clusters. Keyword analysis revealed dementia, quality of life, depression, and cognitive function to be key research themes.

**Conclusion:**

Research on music therapy for older adults is a rapidly expanding and involves strong global collaboration. Our bibliometric findings identify influential countries, institutions, and research networks and indicate that current research generally focuses on demonstrating efficacy for neuropsychiatric symptoms. Future studies should place greater emphasis on understanding mechanisms of action, developing personalized interventions, and evaluating implementation strategies to support the integration of evidence-based music therapy into routine geriatric care.

## Introduction

Music therapy is a well-established nonpharmacological intervention in geriatric care. It improves cognitive function, emotional well-being, and adjustment during transitions to long-term care. Research indicates that music therapy can considerably improve cognitive function and adaptive capacity in older adults. For example, a randomized controlled trial reported that music therapy substantially improved cognitive function and reduced adaptation difficulties in patients with Alzheimer’s disease ( Kayaaslan & Lök, 2025). Music therapy also alleviates depressive symptoms and can serve as a safer alternative to conventional pharmacological treatment, which carries risks related to polypharmacy ( Vaidyanathan & Carroll, 2023). Furthermore, a study reported the beneficial effects of music therapy on diverse health outcomes, including emotional well-being and pain management; however, these effects may decline after intervention ends ( Castillejos & Godoy-Izquierdo, 2021). Music therapy also promotes social interaction and personal expression, both of which support individuals during the transition to long-term care ( Schneible et al., 2023). Thus, many researchers recommend integrating music therapy into geriatric care practices ( Mangiacotti et al., 2024).

Study on music therapy for older adults have reported beneficial effects on cognitive function, mood, and behavioral symptoms, particularly in dementia ( Van der Steen et al., 2025); however, several limitations continue to hinder its systematic application. One major problem is the variability in the designs, interventions, and outcome measures of relevant studies; this variability complicates meta-analyses and limits the development of standardized methods for evaluating music therapy interventions ( Van der Steen et al., 2025). Additionally, large-scale, longitudinal randomized controlled trials evaluating the long-term efficacy and cost-effectiveness of music therapy are generally lacking, with a particular paucity of trials comparing active music therapy with receptive listening interventions ( Fang et al., 2017). Moreover, many studies do not sufficiently distinguish between dementia subtypes or account for individuals’ musical backgrounds and cultural contexts, which has limited the development of personalized interventions ( Baird & Samson, 2015). The mechanisms underlying the effects of music therapy—whether they are neurological, psychosocial, or a combination of both—remain inadequately understood. Additional research integrating biomarkers with psychosocial measures is therefore required to clarify these mechanisms ( Särkämö, 2018). A substantial translational gap also persists between clinical findings and practical, scalable application across various care settings, with few institutions providing staff training on music therapy or incorporating it into routine care plans ( McDermott et al., 2013). Because of these limitations, researchers have yet to obtain robust, context-specific evidence of the effectiveness of music therapy for older adults. In addition, they limit the visibility of such therapy in global academic discourse and hinder the development of relevant guidelines. In consideration of these gaps, a bibliometric analysis is warranted to systematically examine the research on music therapy in older adult populations.

Bibliometric analysis helps researchers understand the development of a research field and identify potential directions for future study. Such analysis enables researchers to identify major trends, prominent authors, collaboration networks, and gaps in the literature. It can provide useful insights that support the development of research in a field and encourage broader collaboration within the field. The findings of our study provide insights into current research on music therapy for older adults and reveal directions for future investigations.

## Methods

### Search strategy

We searched the Scopus database to identify relevant publications. Scopus is among the largest global databases of academic literature and includes approximately 24,000 current publications from more than 5,000 publishers. The database indexes peer-reviewed journals, conference proceedings, commercial magazines, and academic book series across multiple disciplines. At the time of our search, Scopus contained approximately 75 million records. The following keywords were used during the literature search: (ALL (“music therapy”) AND ALL (elderly)). Two experts with at least 5 years of experience in conducting systematic reviews verified the search strategy. After reviewing the titles of all retrieved records, they identified relevant articles. Any disagreements were resolved through discussion with an additional author.

### Screening

Only articles that reported original findings and focused on music therapy in older adults were included in the bibliometric analysis. No language restrictions were applied. Letters, editorials, conference abstracts, and book chapters were excluded, whereas research articles and review papers published in peer-reviewed journals were included. Two specialists with experience in bibliometric analysis independently evaluated the articles according to predefined criteria and guidelines ( Martinez, Al-Hussein, & Ahmad, 2019).

### Bibliometric analysis

Bibliometric analysis is a quantitative approach to evaluating scientific research that involves examining publication patterns within a specific field and generating reproducible data. Such information can inform research and policy decisions in the analyzed field ( Mooghali et al., 2011; Pritchard, 1969). Bibliometric analysis enables systematic examination of a research topic and helps identify emerging areas of investigation and patterns in scholarly activity ( Su & Lee, 2010). Such analysis can reveal patterns and trends in research involving music therapy for older adults.

The present study reported the distribution, proportions, and frequencies of publications for each journal that was considered. The classifications, ratios, and frequencies of the publications were also presented. Author-related ratios, frequencies, and percentages were calculated for each country, both individually and collectively. The publication frequency and citation rates for each author and organization were also reported. The research impact of each country, journal, organization, and author was categorized on the basis of the volume of publications from each. All data were generated and visualized using VOSviewer (Leiden University) and Bibliometrix (an R package).

## Results

### Publication output

After screening was complete, the final bibliometric analysis included 5253 papers. Figure 1 illustrates the increase in the volume of the literature related to music therapy for older adults. Table 1 presents the geographical distribution of the 10 leading countries contributing to research on music therapy for older adults. China was the leading contributor, with 705 publications, followed by the United States with 675 publications and the United Kingdom with 309 publications. Italy had the highest ratio of multiple country publications, at 31.5, followed by Australia at 27.9 and Germany at 24.5. Figure 2 illustrates the network of countries that participated in at least five collaborative research initiatives.

**Figure 1.**
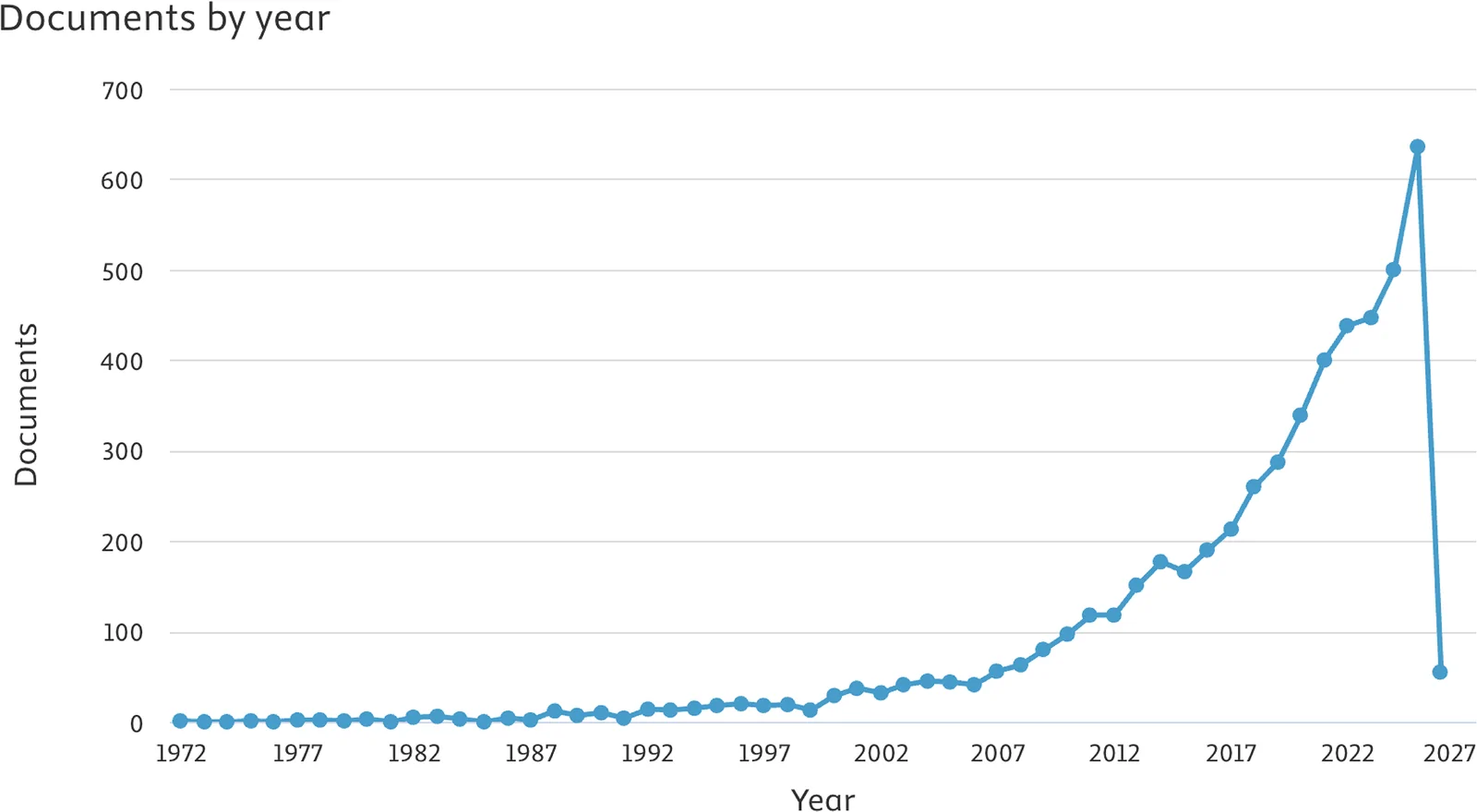
The distribution of articles by year.

**Table 1.** Top 10 countries that published in 1972–2026.

Rank	Country	Articles	Articles %	SCP	MCP	MCP %
1	CHINA	705	13.4	615	90	12.8
2	USA	675	12.9	599	76	11.3
3	UNITED KINGDOM	309	5.9	246	63	20.4
4	AUSTRALIA	222	4.2	160	62	27.9
5	KOREA	220	4.2	207	13	5.9
6	CANADA	182	3.5	141	41	22.5
7	IRAN	177	3.4	142	35	19.8
8	GERMANY	163	3.1	123	40	24.5
9	ITALY	162	3.1	111	51	31.5
10	JAPAN	145	2.8	129	16	11

MCP= multiple country publications; SCP= single country publications.

**Figure 2.**
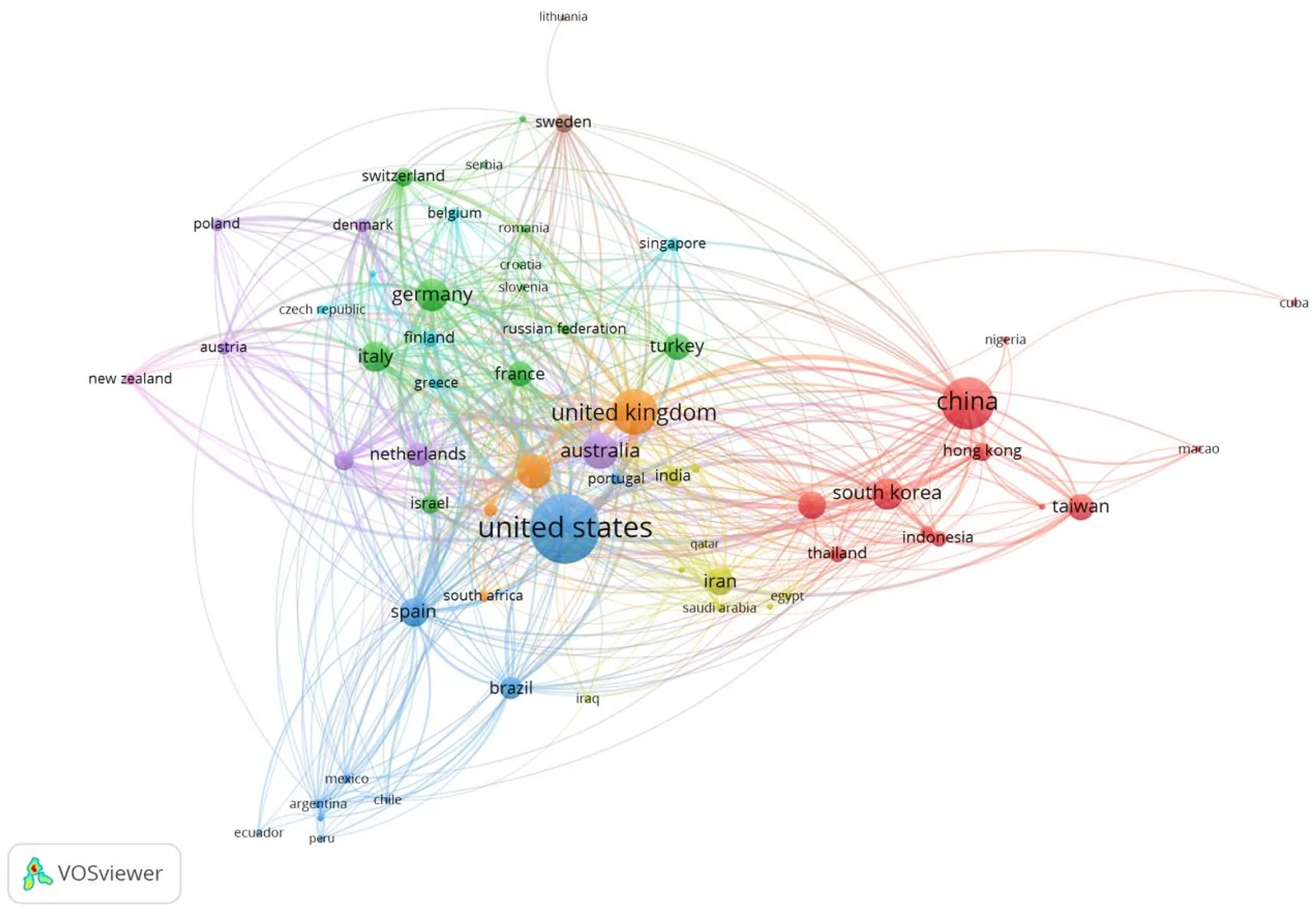
The co-authorship network of country that contributed to the topic.

Supplementary Table 1 presents the 10 leading institutions with the highest publication output in music therapy research involving older adults. The University of Toronto ranked first, with 92 published articles. The University of California followed, with 77 publications, and Anglia Ruskin University produced 58 articles.

VOSviewer was used to conduct a coauthorship analysis to examine the network of countries involved in music therapy research on older adults. The level of collaboration between two countries was determined on the basis of the number of publications they coauthored, with each country being required to contribute at least five articles to the total output. The country-level coauthorship analysis identified six distinct clusters (Supplementary Figure 1).

Supplementary Figure 2 presents an analysis of the 10 most prolific authors in music therapy research involving older adults. Wang Y was the most prolific, with 36 publications on the topic. The influence of the identified authors is further detailed in Supplementary Table 2.

### Co-occurrence analysis of the top 10 keywords

A total of 5253 articles were classified into 6 keyword clusters. Supplementary Table 3 lists the 10 most frequently used keywords in the retrieved articles. Analysis of the co-occurrence of the top 10 keywords identified the main research themes in music therapy research involving older adults ( Figure 3).

**Figure 3.**
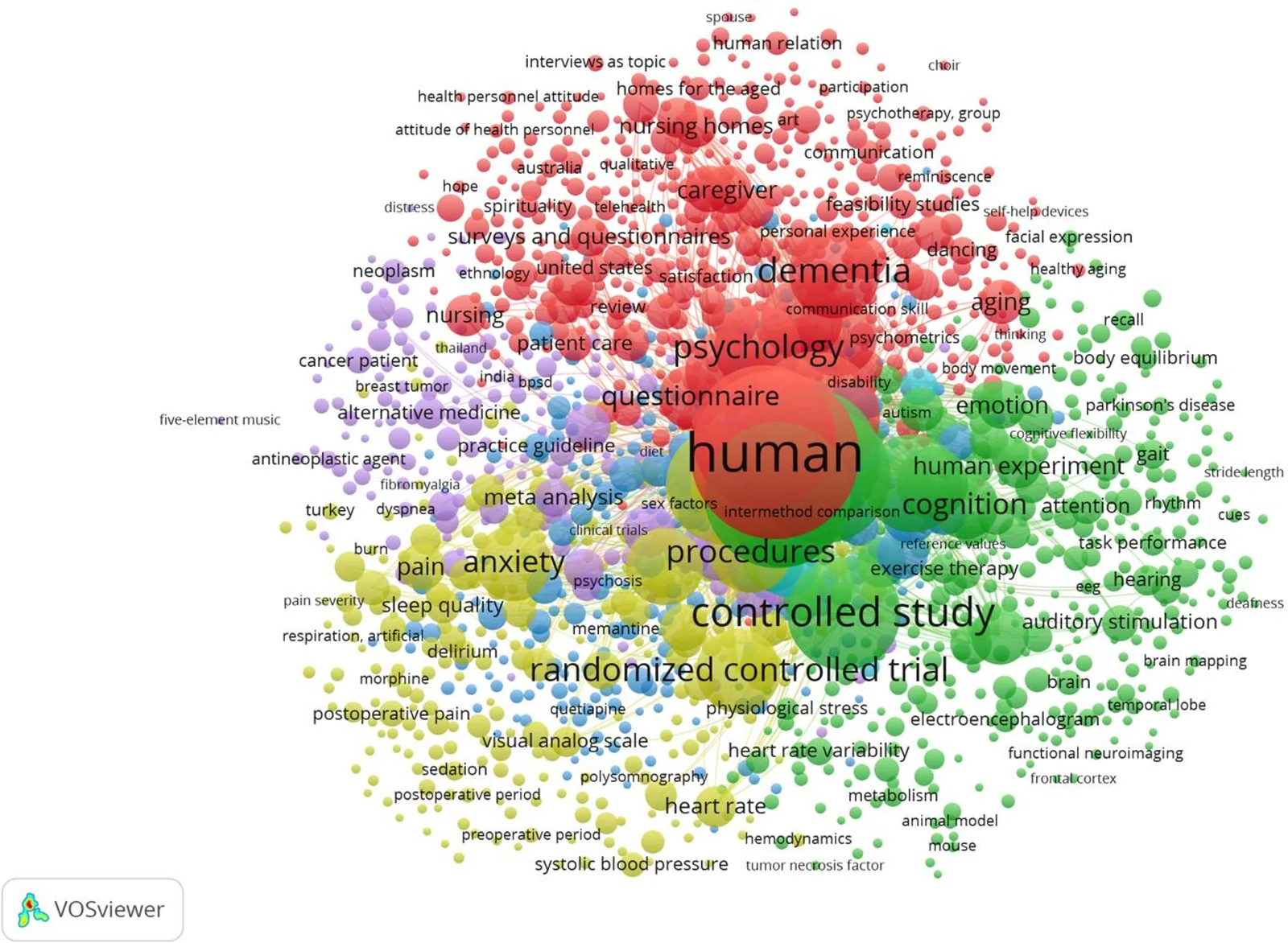
The co-occurrence network of the top 10 keywords in this research. The top 10 keywords were collected and clustered using the visualization application VOSviewer. We used VOSviewer to create a visual network map of the top 10 keywords in six clusters, including their co-occurrence. The colors denote the 6 clusters: red (cluster 1, 458 items), green (cluster 2, 362 items), blue (cluster 3, 264 items), yellow (cluster 4, 247 items), purple (cluster 5, 160 items), and light blue (cluster 6, 8 items). The node label is the keyword, and the number of keywords determines the size of the label. The connection joins two nodes that represent the relationship of two terms.

### Reference cocitation analysis

We conducted an in-depth analysis of the citation structure in music therapy research. Table 2 presents the 10 papers with the highest citation counts. Barr (2013), published in *Critical Care Medicine*, had the highest citation count as of February 9, 2026, with a total of 3045 citations.

**Table 2.** Top 10 high cited articles 1972–2026.

Rank	First author/Journal	DOI	Total Citations	TC per Year
1	BARR J, 2013, CRIT CARE MED	10.1097/CCM.0b013e3182783b72	3045	217.50
2	DEVLIN JW, 2018, CRIT CARE MED	10.1097/CCM.0000000000003299	2810	312.22
3	CHOU R, 2016, J PAIN	10.1016/j.jpain.2015.12.008	2554	232.18
4	NA NA, 2015, ALZHEIMER'S DEMENTIA	10.1016/j.jalz.2015.02.003	1550	129.17
5	JUSLIN PN, 2008, BEHAV BRAIN SCI	10.1017/S0140525X08005293	1339	70.47
6	ZENTNER MR, 2008, EMOTION	10.1037/1528-3542.8.4.494	982	51.68
7	ZHOU JH, 2010, BRAIN	10.1093/brain/awq075	881	51.82
8	BLOOD AJ, 1999, NAT NEUROSCI	10.1038/7299	848	30.29
9	DOODY RS, 2001, NEUROLOGY	10.1212/WNL.56.9.1154	827	31.81
10	GORSKI LA, 2021, J INFUS NURS	10.1097/NAN.0000000000000396	794	132.33

DOI= digital object identifiers; TC= total citation.

## Discussion

This study presents a comprehensive bibliometric analysis of global research on music therapy for older adults, covering 5253 publications. The findings indicate a rapidly growing field characterized by strong international collaboration and leadership from specific countries and institutions. China (705 publications) and the United States (675 publications) are the leading contributors to the scholarly output in this field, suggesting substantial research activity and clinical interest in nonpharmacological interventions for aging populations. This trend is consistent with the global shift toward greater emphasis on developing dementia care strategies ( Livingston et al., 2020). The countries, particularly Italy and Germany, and Australia have the highest levels of international collaboration, as indicated by their high multiple country publication ratios. These findings indicate the presence of an established research network in which international collaboration supports efforts to address complex geriatric challenges and facilitates the exchange of research methods and culturally adapted interventions ( McDermott et al., 2018).

The current study’s institutional analysis identified key research centers contributing to the development of this field. The University of Toronto was the leading institution, with 92 publications, which may reflect substantial research activity in the fields of gerontology and music therapy ( Magee et al., 2017). The high publication output of the University of California system and Anglia Ruskin University highlights the role of specialized research institutions in sustaining research productivity. The constructed coauthorship network, comprising six international clusters, indicates that progress in this field is driven by global collaboration. Such collaboration provides research that represents diverse methodological approaches and clinical contexts, which supports the development of robust and generalizable evidence ( Spiro, Tsiris, & Cripps, 2018).

The cocitation and keyword analyses revealed the intellectual structure and evolving focus of research in this field. The most frequently cited study in this field was that by Barr et al. in *Critical Care Medicine.* This may reflect a broader citation trend toward a focus on the management of delirium and agitation in hospitalized older adults rather than on community-based music therapy. This pattern may indicate that the field has expanded into acute care settings ( Vink et al., 2013). The keyword clusters emphasize commonly studied outcomes such as “dementia,” “quality of life,” “depression,” and “cognitive function,” suggesting that current research largely focuses on managing neuropsychiatric symptoms and enhancing well-being in individuals with cognitive decline. This focus is consistent with the core domains addressed in nonpharmacological treatment guidelines ( Kales, Gitlin, & Lyketsos, 2019). The current analysis also highlights notable gaps in the research on music therapy for older adults: whereas substantial research has investigated intervention efficacy, limited attention has been given to mechanisms of action, cost-effectiveness, and implementation protocols in routine care settings ( Waters et al., 2022).

This bibliometric analysis provides an overview of publication patterns and collaborative networks in the music therapy literature; however, it has several limitations that should be acknowledged. Our reliance on a single database (Scopus) may have resulted in the omission of relevant publications indexed in other databases, which may have affected the geographic representation of the literature. Bibliometric methods are used to measure research productivity and citation influence, not to assess the scientific quality or clinical significance of included studies. In addition, the high citation counts of some articles may reflect their relevance to multiple research areas (e.g., critical care and neurology) rather than their specific contribution to understanding the mechanisms of music therapy in geriatric populations. Bibliometric methods alone cannot distinguish between these possibilities.

## Conclusions

This analysis identifies music therapy research involving older adults as a substantial and expanding field with established global research networks. The findings suggest that early research in this field has primarily focused on demonstrating the effectiveness of music therapy interventions. Future work should build on this collaborative foundation to address key translational challenges. This would involve clarifying the neurobiological and psychosocial mechanisms underlying music therapy through interdisciplinary research that incorporates biomarkers and neuroimaging, developing personalized intervention protocols based on personalized musical and dementia subtype, and conducting pragmatic trials to support sustainable implementation and scalability across various health-care systems. Future research should also translate the growing body of academic evidence in this field into systematic integration of evidence-based music therapy in routine geriatric care.

## Data Availability

No data are associated with this article. Supplementary materials are available in the supplementary data. Zenodo: Supplementary files of Global Research Landscapes and Emerging Trends in Music Therapy for Older Adults: A Comprehensive Bibliometric Analysis: https://doi.org/10.5281/zenodo.21440847 ( Hasan, 2026). This project contains the following extended data:
• Supplementary Figure 1. The co-authorship network of authors who contributed to the topic during 1972 to 2026. • Supplementary Figure 2. Top 10 authors that published in 1972–2026. • Supplementary Table 1. Top 10 institutions that published papers 1972–2026. • Supplementary Table 2. Top 10 authors impact that published in 1972–2026. • Supplementary Table 3. Top 10 author keywords 1972–2026. Data are available under the terms of the Creative Commons Attribution 4.0 International license (CC-BY 4.0).
