# Supplementary material for: Global Research Landscapes and Emerging Trends in Music Therapy for Older Adults: A Comprehensive Bibliometric Analysis
Source: F1000Res. 2026 Aug 24;15:1417. [Version 1] doi: 10.12688/f1000research.187349.1 (PMC13752448; doi:10.12688/f1000research.187349.1)
Supplement: Peer review history

# Reviewer report: Reviewer response for version 1

**DOI:** 10.5256/f1000research.206776.r514949

**Published:** 2026-08-31

**Authors:** Vivi Leona Amelia

**Affiliations:** 1 Universitas Muhammadiyah Purwokerto, Purwokerto, Central Java, Indonesia

***Article: Global Research Landscapes and Emerging Trends in Music Therapy for Older Adults: A Comprehensive Bibliometric Analysis***

***Version: 1***

***DOI: 10.12688/f1000research.187349.1***

**Summary**

 The authors present a bibliometric analysis of 5,253 Scopus-indexed publications on music therapy for older adults, using VOSviewer and Bibliometrix to characterize publication trends, geographic/institutional productivity, collaboration networks, and keyword co-occurrence. The topic is timely and clinically relevant, and the manuscript is generally well organized. However, several methodological and reporting issues should be addressed before the work can be considered fully sound.

- **Is the work clearly and accurately presented, and does it cite the current literature?** Yes

The Introduction is well referenced and frames the clinical rationale clearly.

 There is a subject-verb agreement error in the opening line of the Introduction's second paragraph ("Study on music therapy for older adults have reported...") that should read "Studies... have reported."

- **Is the study design appropriate and is the work technically sound?**

Partly

 Search strategy is narrow. The search string ("music therapy") AND (elderly) may under-capture the literature. Common alternative terms such as "older adults," "aged," "geriatric," or "dementia" (used independently of "elderly") are not included as synonyms/OR terms. Given that the Discussion and keyword clusters explicitly reference "aging," "geriatric," and "dementia" as central themes, the search strategy should be broadened or the rationale for restricting to "elderly" alone should be justified.

 No PRISMA-style flow of record numbers. The Methods state that titles were screened and irrelevant records excluded, but only the final included count (5,253) is reported. The number of records initially retrieved, duplicates removed, and records excluded at each stage should be reported (ideally with a flow diagram), consistent with standard bibliometric/systematic review reporting.

 No inter-rater reliability reported. Two independent screeners are mentioned, but no agreement statistic (e.g., Cohen's kappa) is provided for the screening or the bibliometric-analysis-eligibility stage.

 Figure 1. The annual publication trend shows a sharp rise to ~640 documents followed by an abrupt drop to near zero at what is labeled "2027." Since the search was conducted "through February 2026," this final data point almost certainly reflects an incomplete indexing year mislabeled on the x-axis (or a partial 2026 count plotted as 2027). This should be corrected and explicitly addressed in the text (e.g., noting that the final year is partial and should be interpreted with caution), rather than left to inference.

 Keyword network figure/caption inconsistency. The Methods and Figure 3 caption both describe an analysis of the "top 10 keywords," yet the caption also states the network comprises six clusters totaling 458 + 362 + 264 + 247 + 160 + 8 = 1,499 items. This is inconsistent with a "top 10 keyword" analysis. Please clarify whether the reported figure reflects a full co-occurrence network above a minimum occurrence threshold (with the "top 10" limited to the accompanying frequency table), and correct the text/caption/Methods accordingly.

 Reference cocitation analysis raises a relevance concern. The most-cited paper identified (Barr et al., 2013, Critical Care Medicine — ICU pain/agitation/delirium management guidelines) does not appear to be substantively about music therapy. The authors acknowledge in the Discussion that high citation counts "may reflect... relevance to multiple research areas... rather than their specific contribution to... music therapy," which is a reasonable caveat, but this also raises a more fundamental question: were these highly-cited records actually retrieved because they matched the search terms on music therapy and older adults, or are they appearing due to a citation-network artifact? This should be clarified, since it bears on whether the underlying corpus of 5,253 papers is precise (i.e., whether the search strategy in point 1 above is producing false positives).

- **Are sufficient details of methods and analysis provided to allow replication?**

Partly: The search string, database, and software are specified, which is good. However, VOSviewer clustering parameters (e.g., minimum keyword occurrence threshold, resolution parameter) are not fully reported for the keyword co-occurrence analysis, only for the country coauthorship analysis (minimum 5 publications/country). These should be specified for full reproducibility.

- **If applicable, is the statistical analysis and its interpretation appropriate?**

Yes: I independently checked the arithmetic in Table 1 (SCP + MCP = Articles; MCP% = MCP/Articles) for all 10 countries, and all values are internally consistent.

- **Are all the source data underlying the results available to ensure full reproducibility?**

Yes: Supplementary materials are deposited on Zenodo per the Data Availability statement. I was not able to independently verify the supplementary files as part of this review; the editors/authors should confirm the deposited files match the version under review.

- **Are the conclusions drawn adequately supported by the results?**

Partly: The main descriptive conclusions (leading countries, institutions, collaboration patterns, keyword themes) are reasonably supported by the reported data. However, conclusions about "key research themes" and "mechanisms of action gaps" would be strengthened once the search-strategy and citation-relevance concerns above are addressed, since they affect confidence in the underlying corpus.

**Other minor issues**

 Funding/author-role inconsistency. The Grant Information statement declares "no grants were involved in supporting this work," yet the Author Roles list "Funding Acquisition" as a contribution for two authors (Phanphairoj K and Hasan F). Please clarify or correct this apparent contradiction.

 Consider noting explicitly in the Limitations paragraph that the search terminology may have contributed to under-representation of relevant literature indexed under alternative terms.

**Approval Status**

 Approved with Reservations

 The topic is valuable and the overall bibliometric approach is appropriate, but the search-strategy scope, the Figure 1 year-axis anomaly, the Figure 3/Methods "top 10 keyword" inconsistency, the questionable relevance of the top-cited paper, and the funding/author-role discrepancy should be addressed before the article can be considered methodologically sound.

Is the work clearly and accurately presented and does it cite the current literature?

Yes

If applicable, is the statistical analysis and its interpretation appropriate?

Yes

Are all the source data underlying the results available to ensure full reproducibility?

Yes

Is the study design appropriate and is the work technically sound?

Partly

Are the conclusions drawn adequately supported by the results?

Partly

Are sufficient details of methods and analysis provided to allow replication by others?

Partly

Reviewer Expertise:

Nursing, Mental Health, Sleep study

I confirm that I have read this submission and believe that I have an appropriate level of expertise to confirm that it is of an acceptable scientific standard, however I have significant reservations, as outlined above.

# Response

**Published:** 2026-09-07

**Authors:** faizul hasan

**Affiliations:** faculty of nursing, Chulalongkorn University, Bangkok, Bangkok, Thailand

Reviewer:

 Dr Vivi Leona Amelia, Universitas Muhammadiyah Purwokerto, Purwokerto, Central Java, Indonesia

 Summary

 The authors present a bibliometric analysis of 5,253 Scopus-indexed publications on music therapy for older adults, using VOSviewer and Bibliometrix to characterize publication trends, geographic/institutional productivity, collaboration networks, and keyword co-occurrence. The topic is timely and clinically relevant, and the manuscript is generally well organized. However, several methodological and reporting issues should be addressed before the work can be considered fully sound.

 Response: We sincerely thank the reviewer for the thoughtful and constructive feedback. We have addressed each point carefully, making conservative revisions where appropriate, and have clarified methodological choices to avoid misinterpretation. All changes are highlighted in red in the revised manuscript.

 Is the work clearly and accurately presented, and does it cite the current literature? Yes

 The Introduction is well referenced and frames the clinical rationale clearly.

 There is a subject-verb agreement error in the opening line of the Introduction's second paragraph ("Study on music therapy for older adults have reported...") that should read "Studies... have reported."

 Response: We have revised it.

 Is the study design appropriate and is the work technically sound?

 Partly

 Search strategy is narrow. The search string ("music therapy") AND (elderly) may under-capture the literature. Common alternative terms such as "older adults," "aged," "geriatric," or "dementia" (used independently of "elderly") are not included as synonyms/OR terms. Given that the Discussion and keyword clusters explicitly reference "aging," "geriatric," and "dementia" as central themes, the search strategy should be broadened or the rationale for restricting to "elderly" alone should be justified.

 Response: We acknowledge the reviewer’s concern. However, we deliberately used the term "elderly" as the sole population descriptor to maintain focus and consistency with the core clinical population of interest in this bibliometric mapping exercise. While we agree that terms like "older adults," "aged," and "geriatric" appear in the literature, our preliminary scoping indicated that "elderly" is the most frequently used MeSH-compatible term in Scopus for this age group and yielded a sufficiently large and representative corpus (5,253 records) for robust bibliometric analysis.

 We have now added a clear justification in the Methods section (lines 110–115) stating that the search was not intended to be exhaustive like a systematic review, but rather to capture a coherent dataset for trend analysis. We also acknowledge this as a limitation in the Discussion (lines 450–455), noting that alternative terms may have enriched the dataset, but that our primary aim was to map dominant patterns within the "elderly"‑focused literature.

 No PRISMA-style flow of record numbers. The Methods state that titles were screened and irrelevant records excluded, but only the final included count (5,253) is reported. The number of records initially retrieved, duplicates removed, and records excluded at each stage should be reported (ideally with a flow diagram), consistent with standard bibliometric/systematic review reporting.

 Response: We appreciate the reviewer’s suggestion, but we respectfully clarify that this is a bibliometric analysis, not a systematic review. PRISMA guidelines are not mandatory for descriptive bibliometric studies, and their application can imply a level of evidence synthesis that goes beyond our scope.

 No inter-rater reliability reported. Two independent screeners are mentioned, but no agreement statistic (e.g., Cohen's kappa) is provided for the screening or the bibliometric-analysis-eligibility stage.

 Response: We agree that reporting inter-rater reliability is good practice. However, in bibliometric studies, screening is typically a binary inclusion/exclusion based on broad topic relevance, and disagreements are resolved by consensus rather than by formal statistical agreement. To address the reviewer’s point, we have now calculated Cohen’s kappa for the screening stage on a random sample of 200 records, which yielded κ = 0.89 (95% CI: 0.84–0.94), indicating almost perfect agreement. This information has been added to the Methods (lines 130–135) and to the Results (lines 210–212). We thank the reviewer for prompting this added rigour.

 Figure 1. The annual publication trend shows a sharp rise to ~640 documents followed by an abrupt drop to near zero at what is labeled "2027." Since the search was conducted "through February 2026," this final data point almost certainly reflects an incomplete indexing year mislabeled on the x-axis (or a partial 2026 count plotted as 2027). This should be corrected and explicitly addressed in the text (e.g., noting that the final year is partial and should be interpreted with caution), rather than left to inference.

 Response: We thank the reviewer for identifying this critical issue. After re-examining the raw data exported from Scopus, we confirm that the year label appearing as "2027" on the x-axis of Figure 1 is not a typographical error but rather a reflection of how Scopus indexes publications with online-first or ahead-of-print status.

 Keyword network figure/caption inconsistency. The Methods and Figure 3 caption both describe an analysis of the "top 10 keywords," yet the caption also states the network comprises six clusters totaling 458 + 362 + 264 + 247 + 160 + 8 = 1,499 items. This is inconsistent with a "top 10 keyword" analysis. Please clarify whether the reported figure reflects a full co-occurrence network above a minimum occurrence threshold (with the "top 10" limited to the accompanying frequency table), and correct the text/caption/Methods accordingly.

 Response: We apologise for this ambiguity. The confusion arose because we presented both a frequency table of the top 10 most occurring keywords and a full co‑occurrence network based on a minimum occurrence threshold of 5 and a minimum cluster size of 3. The network includes all 1,499 keywords meeting that threshold, not only the top 10. We have now revised the Methods (lines 145–150) and the Figure 3 caption to clearly differentiate between the top‑10 frequency list and the full network visualisation. The caption now reads: "Figure 3: Keyword co‑occurrence network (threshold: minimum 5 occurrences; 6 clusters; 1,499 total items). The accompanying frequency table lists the top 10 most frequent keywords." We also corrected the main text accordingly.

 Reference cocitation analysis raises a relevance concern. The most-cited paper identified (Barr et al., 2013, Critical Care Medicine — ICU pain/agitation/delirium management guidelines) does not appear to be substantively about music therapy. The authors acknowledge in the Discussion that high citation counts "may reflect... relevance to multiple research areas... rather than their specific contribution to... music therapy," which is a reasonable caveat, but this also raises a more fundamental question: were these highly-cited records actually retrieved because they matched the search terms on music therapy and older adults, or are they appearing due to a citation-network artifact? This should be clarified, since it bears on whether the underlying corpus of 5,253 papers is precise (i.e., whether the search strategy in point 1 above is producing false positives).

 Response: We thank the reviewer for raising this critical point. After verification, we confirm that Barr et al. (2013) was not retrieved directly by our search string. It appears in the reference cocitation analysis because it was frequently cited by the papers in our corpus, not because it matched the search terms. This is a known and expected feature of cocitation mapping: highly cited foundational or guideline papers from adjacent fields often emerge as nodes even if they are not themselves about the primary topic. To avoid any misinterpretation, we have now:

- Added a clear explanation in the Methods (lines 160–165) that cocitation analysis includes all references cited by the retrieved papers, regardless of whether they directly match the search string.
- In the Results (lines 280–285), we explicitly state that Barr et al. (2013) is a citation‑network artifact and does not imply that it was part of the original 5,253 records.
- In the Discussion (lines 470–475), we strengthened the caveat that highly cited external papers may inflate perceived influence and should not be misconstrued as direct evidence of music therapy research.

Are sufficient details of methods and analysis provided to allow replication?

 Partly: The search string, database, and software are specified, which is good. However, VOSviewer clustering parameters (e.g., minimum keyword occurrence threshold, resolution parameter) are not fully reported for the keyword co-occurrence analysis, only for the country coauthorship analysis (minimum 5 publications/country). These should be specified for full reproducibility.

 Response: We agree and have now added the missing parameters to the Methods (lines 140–148). For keyword co‑occurrence, we used a minimum occurrence threshold of 5, a resolution parameter of 1.0, and a minimum cluster size of 3. For country co‑authorship, the minimum was 5 documents per country, with the same resolution and cluster size. These details are now fully reported.

 If applicable, is the statistical analysis and its interpretation appropriate?

 Yes: I independently checked the arithmetic in Table 1 (SCP + MCP = Articles; MCP% = MCP/Articles) for all 10 countries, and all values are internally consistent.

 Response: We sincerely thank the reviewer for the careful verification of the arithmetic in Table 1. We appreciate the time and diligence taken to independently check the internal consistency of the SCP, MCP, and MCP% calculations for all 10 countries. We confirm that all values in Table 1 are mathematically consistent, and no corrections are required. We have retained Table 1 exactly as presented in the original submission.

 Are all the source data underlying the results available to ensure full reproducibility?

 Yes: Supplementary materials are deposited on Zenodo per the Data Availability statement. I was not able to independently verify the supplementary files as part of this review; the editors/authors should confirm the deposited files match the version under review.

 Response:

 We thank the reviewer for acknowledging the data availability statement and for highlighting the importance of verifying the deposited files. We respectfully note that, as authors, we are unable to independently confirm that the Zenodo files exactly match the version under review, as we did not personally perform this verification prior to submission.

 Are the conclusions drawn adequately supported by the results?

 Partly: The main descriptive conclusions (leading countries, institutions, collaboration patterns, keyword themes) are reasonably supported by the reported data. However, conclusions about "key research themes" and "mechanisms of action gaps" would be strengthened once the search-strategy and citation-relevance concerns above are addressed, since they affect confidence in the underlying corpus.

 Response: We agree that the conclusions should be tempered. In light of the clarifications made above, we have revised the Conclusions section (lines 580–590) to be more conservative, emphasising that the identified themes are derived from the current corpus and may not represent the full spectrum of music therapy research in older adults. We also highlight the need for future systematic reviews to confirm these thematic patterns with broader search strategies.

 Other minor issues

 Funding/author-role inconsistency. The Grant Information statement declares "no grants were involved in supporting this work," yet the Author Roles list "Funding Acquisition" as a contribution for two authors (Phanphairoj K and Hasan F). Please clarify or correct this apparent contradiction.

 Response: We appreciate the reviewer’s sharp eye. This was an administrative oversight in the author roles section. The authors listed under "Funding Acquisition" contributed to securing institutional support (e.g., internal departmental funds) but not external grants. Upon review of our funding records and author contribution documentation, we confirm that Kanjanee Phanphairoj received internal institutional funding from Chulalongkorn University to support this research. We have now revised the manuscript to accurately reflect this funding source and resolve the apparent contradiction.

 Consider noting explicitly in the Limitations paragraph that the search terminology may have contributed to under-representation of relevant literature indexed under alternative terms.

 Response: We have added a clear limitation statement (lines 455–458): "Our use of the single term 'elderly' may have under‑represented studies indexed under alternative terms such as 'older adults' or 'geriatric.' This was a deliberate choice for thematic coherence, but readers should interpret the findings as reflective of the 'elderly'‑focused literature rather than the entire field of music therapy in aging."

 Approval Status

 Approved with Reservations

 The topic is valuable and the overall bibliometric approach is appropriate, but the search-strategy scope, the Figure 1 year-axis anomaly, the Figure 3/Methods "top 10 keyword" inconsistency, the questionable relevance of the top-cited paper, and the funding/author-role discrepancy should be addressed before the article can be considered methodologically sound.

 Response: I appreciate your acknowledgement.
